# The lncRNA Connection Between Cellular Metabolism and Epigenetics in Trained Immunity

**DOI:** 10.3389/fimmu.2018.03184

**Published:** 2019-01-29

**Authors:** Ezio T. Fok, Laurianne Davignon, Stephanie Fanucchi, Musa M. Mhlanga

**Affiliations:** ^1^Division of Chemical, Systems & Synthetic Biology, Department of Integrative Biomedical Sciences, Faculty of Health Sciences, Institute of Infectious Disease & Molecular Medicine, University of Cape Town, Cape Town, South Africa; ^2^Gene Expression and Biophysics Group, ERA, CSIR Biosciences, Pretoria, South Africa; ^3^Gene Expression and Biophysics Unit, Instituto de Medicina Molecular, Faculdade de Medicina Universidade de Lisboa, Lisbon, Portugal

**Keywords:** trained immunity, epigenetics, metabolism, long non-coding, inflammation, nuclear architecture, immunological “memory”

## Abstract

Trained immunity describes the ability of innate immune cells to form immunological memories of prior encounters with pathogens. Recollection of these memories during a secondary encounter manifests a broadly enhanced inflammatory response characterized by the increased transcription of innate immune genes. Despite this phenomenon having been described over a decade ago, our understanding of the molecular mechanisms responsible for this phenotype is still incomplete. Here we present an overview of the molecular events that lead to training. For the first time, we highlight the mechanistic role of a novel class of long non-coding RNAs (lncRNAs) in the establishment and maintenance of discrete, long lasting epigenetic modifications that are causal to the trained immune response. This recent insight fills in significant gaps in our understanding of trained immunity and reveals novel ways to exploit trained immunity for therapeutic purposes.

## Introduction

Immune memory is the ability of the immune system to recognize antigens from prior exposure to pathogens in order to elicit a rapid and effective immune response. The ability to form immune memory has been solely ascribed to the adaptive arm of the immune system and specifically described as cellular and humoral immunity. Together, these different modalitlies of adaptive immunity refer to memory encoded in highly specific epitope recognition, leading to the reactivation and expansion of T and B-cell reservoirs. Upon reexposure to a pathogen, T-cell receptors will recognize the pathogen and potentiate the subsequent immune response. In the case of B-cells, a prior exposure will result in the formation of antibodies. Thus, these cells of the adaptive immune system preserve a memory of prior exposures in expanded T and B-cell populations, with specific cell surface receptors or the production of antibodies eliciting immunological responses that are vastly shaped by past engagements with pathogens and vaccines. However, recent observations have revealed that innate immune cells of the myeloid lineage, particularly monocytes and macrophages, also have the capacity to form immune memories (1–7). Indeed, in many plants and invertebrates which lack an adaptive immune system, evidence of acquiring long-term immune memory to provide protection against secondary infections has existed for quite some time (8–11). Perhaps the earliest observation of this innate immune memory in mammals was made in athymic mice that were vaccinated with an attenuated strain of *Candida albicans*. Surprisingly, these immunocompromised mice displayed immunological protection when later challenged with a virulent strain of *C. albicans* or the infectious bacteria *Staphylococcus aureus* (12, 13). Further to gaining this protection independently of T-cells, it was shown that the activation of macrophages and the strong induction of cytokines was necessary to elicit this response, both of which are part of innate immune function (12, 14). In humans, it has been observed that children in West Africa who were vaccinated with BCG (Bacille Calmette-Guérin) attained improved non-specific immunological protection and improved survival against infection (15). This broad protection was later attributed to the significantly enhanced production of cytokines by myeloid cells (16). Strikingly, the population of cells that were imbued with these enhanced protective capabilities persisted for over a month, revealing a long lasting innate immune memory associated with vaccination.

**Figure 1:**
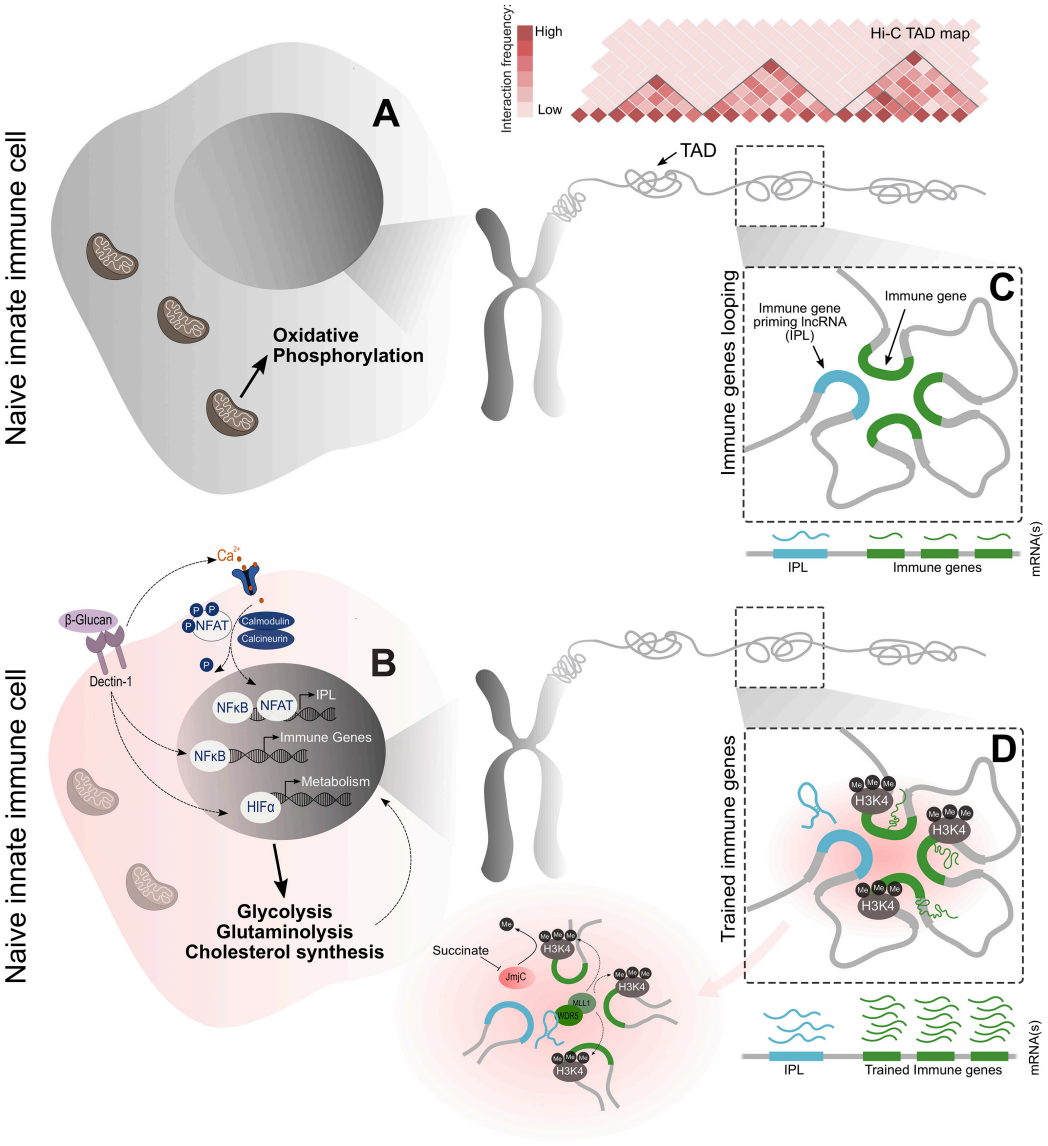
An overview of the molecular events that lead to the establishment of the epigenetic memory underlying trained immunity. **(A,B)** Training begins with the primary exposure of monocytes to β-glucan. This activates the Dectin-1 receptor and calcium-dependent NFAT signaling to initialize transcriptional programs related to immunity and metabolism. The metabolic signaling results in changes to glucose, glutamine, and cholesterol metabolism, which together, supply the metabolites and co-factors essential for the induction and maintenance of the epigenetic changes that are causal to the trained phenotype. NFAT signaling induces the transcription of the newly identified IPLs within immune TADs. **(C,D)** These lncRNAs facilitate the transcriptional priming of the trained immune genes by recruiting the WDR5/MLL histone methyltransferase complex and exploiting the spatial proximity of immune genes to discreetly deposit the H3K4me3 epigenetic mark on their promoters. These events culminate in a more powerful pro-inflammatory response through the enhanced transcription of trained immune genes upon secondary stimulation.

Subsequently, it has been shown that this enhanced cross-protection (termed trained immunity) can be induced in myeloid cells by a variety of stimuli, including cytokines, fungal chitins and bacterial, and metazoan antigens (16–19). In the laboratory setting, this phenotype has been successfully recapitulated in a standard cellular model, in which monocytes are educated by pre-exposure to β-glucan-a major component of the cell wall of *C. albicans*. When these cells were subsequently challenged with a simulated bacterial infection, an enhanced inflammatory response characterized by the increased transcriptional activity of innate immune genes was observed (4, 6). With the establishment of this model, the phenotype of trained immunity in monocytes and macrophages was studied much more closely. Hallmark characteristics associated with the induction of this training include the activation of various pathogen recognition signaling pathways, a shift in cellular metabolism towards a glycolytic state and extensive epigenetic alterations throughout the genome (4, 6, 20, 21). These events culminate in the observed substantial increase in transcriptional activity of the innate immune genes when restimulated. Integral to this is the discrete accumulation of histone modifications which allow for the rapid and robust transcription of immune genes. In the initial training phase, lysine 27 of histone 3 (H3K27) and lysine 4 of histone 3 (H3K4) at the promoters of trained immune genes are rapidly acetylated and trimethylated, respectively (6). These epigenetic histone modifications are positively associated with transcriptional output and are responsible for remodeling the local chromatin into an open and accessible state to facilitate the loading of transcriptional machinery. Upon the removal of the training stimulus, it was observed that H3K27Ac was lost over time, but the H3K4me3 occupancy did not return to the basal state and remained accumulated on the chromatin. It is this long-lived accumulation of H3K4me3 that has been implicated in priming the immune genes and establishing the epigenetic memory required for a trained immunological response (Figure 1) (4, 6, 22).

The long-term persistence and systemic acquisition of this enhanced immunological response, as observed with BCG vaccination, implicates the establishment of a long-lived reservoir of trained cells. In the case of tissue-resident macrophages, such as alveolar macrophages, these cells turn over slowly and are capable of self-renewing independently of circulating monocytes (23). It was recently demonstrated that these alveolar macrophages are trained by contact with CD8 T-cells and IFN-γ, producing an enduring population of trained, lung-resident, macrophages (23). In contrast, circulating bone-marrow derived monocytes have a relatively short lifespan and therefore cannot maintain the trained phenotype systemically. In another recent study, it was demonstrated that BCG vaccination skews haematopoietic stem cell (HSC) and multipotent progenitor (MPP) differentiation to favor myelopoiesis (24). The macrophages that are derived from these pre-exposed progenitors displayed epigenetic and transcriptomic changes that conferred enhanced immunological responses, resulting in increased protection against *Mycobacterium tuberculosis* infection (24). This demonstrated that immune training can occur at the level of the progenitors, creating a source of long-lived immunologically trained cells that can transmit their phenotype (and its associated epigenetic profile) to their terminally differentiated progeny. In the case of acquiring trained immunity from infection, inter-cellular signaling mechanisms are responsible for propagating the trained phenotype from a few initially exposed immune cells at the site of infection, to the systemic level. At the site of pathogen exposure, neutrophils produce neutrophil extracellular traps (NETs) to powerfully induce IL1-β expression, which is a known inducer of trained immunity (16, 25). The elevated levels of circulatory IL1-β may then penetrate the bone marrow to train the myeloid progenitors in the absence of direct pathogen exposure. In this way, the primary training stimulus at the site of infection is amplified by IL1-β paracrine signaling, so that the trained phenotype can be transmitted to the myeloid progenitors for the establishment of a long-lasting, heritable, and systemic trained immune response. However, the molecular mechanism of how these self-renewing cells maintain the trained epigenetic profile, through many generations, remains opaque.

In this review, we discuss the molecular mechanisms that underlie trained immunity, with specific attention to how discrete epigenetic changes manifest at the promoters of trained immune genes. Within the last decade, efforts to decode the function of the genome have revealed the pivotal roles of nuclear architecture and lncRNAs in the epigenetic regulation of gene transcription (26, 27). We highlight recent published findings into the role that these genomic elements have in the mechanism of trained immunity. We contextualize these findings by discussing the well-established signaling pathways and metabolic changes associated with trained immunity, which ultimately, converge in the nucleus to drive significant epigenetic and transcriptional alterations. We envision that this latest piece in the puzzle will be important in shaping our emerging understanding of the field. With a more complete overview of the molecular processes leading to training, a clearer picture of the relationship between the various hallmarks associated with trained immunity can now be revealed. Furthermore, this deepened and integrated understanding of the molecular mechanisms underpinning trained immunity may prove to be highly valuable in any endeavors to exploit it for therapeutic purposes.

## The Role of Receptors, Signaling Cascades and Transcription Factors

Innate immune memory formation begins with the activation of pathogen recognition receptors (PRRs), such as Toll-like receptors (TLRs), C-type lectin receptors (CLRs), NOD-like receptors (NLRs), and RigI-helicases. The discovery of these types of receptors have challenged the dogma that the innate immune system is completely non-specific, as these receptors are able to activate innate immune cells in a specific manner through the recognition of conserved pathogen-associated molecular patterns (PAMPs) (28). The activation of these receptors by a primary stimulant is an important first step in the process of innate immune memory formation. The signals captured by the PRRs traverse the cytosol via different signaling cascades, which lead to transcription factor-dependent activation of specific genes that allow for the cell to execute a dampened (tolerized) or enhanced (trained) immune response when it is exposed to the secondary stimulant.

Monocytes and macrophages have been established as the standard cellular models with which to study trained immunity. This has been classically assayed in monocytes by pre-exposure to fungal or yeast β-glucans. This stimulates the CLR type, dectin-1 receptor, which has been shown by pathway analysis of RNA-seq and ChIP-seq experiments, to activate pathways associated with inflammation and metabolism (Figure 1B) (4, 6, 20). Along the inflammatory signaling axis, dectin-1 signals elicit a weak pro-inflammatory response via NF-κB translocation into the nucleus, through the Syk/Src, and Raf-1 pathways (29). β-glucan/dectin-1 signaling has also been shown to activate the transcription of immune genes via the calcium-dependent family of transcription factors called NFATs (30). Mechanisms linking the immunological signals induced by the primary stimulus to the establishment of epigenetic memory, which forms the basis of trained immunity, until now have been unclear. Prior to recent findings it was thought that this may simply be an unavoidable side-effect of activating pathogen associated signaling pathways. Recent data from the Mhlanga laboratory (31) has shed light on these questions and others. In a recent study it has been shown that blocking NFAT signal transduction significantly hampers training, highlighting the importance of this initial activation of the immune genes (31).

Along the metabolic signaling axis, genes associated with a glycolytic state are enriched after β-glucan/dectin-1 signaling. Central to this is the activation of the Akt-mTOR-HIF1α pathway which drives the essential metabolic shift from oxidative phosphorylation to aerobic glycolysis in trained macrophages (Figures 1A,B). The importance of this glycolytic shift has been frequently demonstrated, with any abrogation of this pathway being detrimental to acquiring trained immunity (20). Furthermore, similar signaling outcomes are observed with other training stimuli. BCG induces training by signaling through the NOD2 receptor and via the RIP2 pathway, to similarly elicit a NF-κB-mediated immune response (32, 33). Metabolically, BCG trained macrophages also activate the Akt-mTOR-HIF1α pathway (34). The conserved activation of both NF-κB immune signaling and Akt-mTOR-HIF1α metabolic signaling, when induced by different stimuli and recognition receptors, highlights the importance of these signaling events in establishing the trained phenotype.

## The Role of Metabolism

Our conventional understanding of how cellular signaling regulates metabolism involves the coupling of the metabolic machinery to the growth and survival needs of the cell. However, with the advancement in the fields of immunology and metabolism within the last decade, it has been revealed that the two are also highly interconnected. There is now increasing evidence that links cellular metabolism to immune function. For example, macrophages activated to become pro-inflammatory favor a glycolytic metabolic state over their anti-inflammatory counterparts, which primarily make use of oxidative phosphorylation (35). Similarly, β-glucan trained monocytes exhibit increased glycolytic metabolism of glucose (Figures 1A,B) (20). However, the mechanisms that link the metabolic state associated with trained immunity to the establishment of the epigenetic changes that are responsible for enhanced gene transcription, have not been clear until quite recently.

The most apparent reason for the glycolytic shift in trained monocytes or macrophages is to meet the increased bioenergetic requirements that the cell anticipates for mounting an enhanced secondary inflammatory response (20). This metabolic switch seems to be counter-intuitive, as oxidative phosphorylation is much more efficient at ATP generation. However, the glycolytic pathway for energy production can be rapidly activated through the induction of the associated enzymes, whereas oxidative phosphorylation relies on mitochondrial biogenesis, which occurs on a much slower timescale. Furthermore, this also makes glycolysis rapidly scalable, as the cell can quickly ramp up ATP production by increasing the expression of the glycolytic enzymes through epigenetic mechanisms (6, 35). Thus, the glycolytic metabolism of glucose elevates the bioenergetic readiness of a trained cell in order to decrease the kinetic lag associated with generating the activation energy needed for enhanced transcription and inflammation. This results in a response that more closely resembles digital activation. With this, the cellular environment of a trained macrophage is metabolically geared toward a high energy state that allows for it to quickly elicit a significantly more powerful inflammatory response when stimulated again.

In the trained state, it has been shown that the alterations in cellular metabolism are not exclusive to shifts in glucose metabolism. Instead, by correlating pathway analysis from RNA-seq data with careful metabolomic measurements, it was revealed that changes in glutamine and cholesterol metabolism are also crucial for training (Figure 1B) (21). Accompanying these metabolic alterations, is the production and accumulation of various intermediary metabolites which serve as important substrates and co-factors for the activity of chromatin writers (such as histone methyltransferases and acetyltransferases) and erasers (such as histone demethylases and deacetlyases). The regulation of the activity of these enzymes, through the metabolic control of the availability of their substrates and co-factors, provides a strong correlative link between cellular metabolism and the epigenetic regulation of gene transcription (21) with a direct mechanistic link remaining unelucidated.

Causal to the enhanced inflammatory response in trained immunity is the increased deposition of histone marks that are positively correlated with transcription at the promoters of key immune genes. The promoters of trained immune genes are enriched for H3K4me3 and H3K27Ac (4, 6). Both these marks are permissive for gene transcription. H3K27Ac is associated with increased chromatin openness, facilitating transcription factor binding at promoter regions and active enhancer elements to positively regulate transcriptional activity (36, 37). H3K4me3 is hypothesized to increase the local hydrophobicity of the chromatin, allowing for liquid-liquid phase separated transcription factors to engage with the DNA in a more energetically favorable phase transition in the aqueous environment of the nucleus (38–40). This aids in the preparatory loading of transcriptional machinery onto promoters, so that they are suspended in a transcriptionally poised state while they await for signals that rapidly induce a shift into active transcription (41, 42). Therefore, these epigenetic marks are particularly important for the robust and rapid activation of the immune genes for a successful inflammatory response to be mounted (43). The changes in cellular metabolism observed in trained cells favor these histone modifications, allowing for the trained immune genes to be primed for enhanced gene transcription.

The deposition of H3K27Ac by histone acetyltransferases relies on the availability of acetyl-CoA as a substrate. As a result, the abundance of acetyl-CoA impacts histone acetylation and gene transcription (44, 45). Glucose-derived pyruvate can be converted to acetyl-CoA and serve as a source of this acetyl donor (35, 44). In the case of trained monocytes and macrophages, the increased glucose uptake and metabolism ensures that a sufficient cellular concentration of acetyl-CoA is maintained. Supporting the accumulation of histone acetylation is the production of lactate, which is a product of glycolytic metabolism and has been shown to inhibit histone deacetylase activity (46). Despite primary energy production being shifted toward glycolysis, the activity of the tricarboxylic acid (TCA) cycle remains highly active under trained conditions (47). The increase in glutamine metabolism feeds into the TCA cycle, via glutamate, to maintain its functionality. Furthermore, it was recently shown that β-glucan enhances the expression of succinate dehydrogenase, a key enzyme in driving the TCA cycle (47). As a result of this increased TCA cycle activity, acetyl-CoA is also produced as an intermediary metabolite in the pathway for cholesterol synthesis, which is known to be upregulated in trained monocytes and macrophages (21, 44). Moreover, metabolomic measurements have shown that the continued activity of the TCA cycle in trained monocytes results in the accumulation of succinate and fumarate (21). Both these metabolites act as competitive antagonists to α-ketoglutarate, which is a cofactor for lysine demethylases (48, 49). More specifically, succinate and fumarate are able to inhibit the function of the jumonji (JMJ)/KDM5 family of histone lysine demethylases which, importantly, are the only enzymes that can actively remove lysine trimethylation, such as the activating H3K4me3 and the repressive H3K27me3 marks (38, 50). Thus, with the accumulation of these metabolites during training, the transcriptionally poised state of trained immune genes is maintained via the preservation of the H3K4me3 mark (Figures 1B,D). Furthermore, succinate and fumarate have been shown to stabilize HIF1α, reinforcing the activation of the metabolic pathways associated with training (20, 51, 52). Interestingly, the exposure of monocytes to fumarate alone was able to partially recapitulate the trained phenotype, highlighting the powerful role that these metabolites from the TCA cycle have in shaping the epigenetic landscape that underlies trained immunity (21).

Collectively, the alteration of cellular metabolism in immune training prepares the cell for the subsequent stimulation, such that it can mount a much stronger pro-inflammatory response. These metabolic changes ensure that the bioenergetic requirements for this are rapidly met by adopting a more suitable method of glucose metabolism for energy production. Furthermore, the changes in glucose, glutamine, and cholesterol metabolism collectively create a stockpile of metabolites that positively influence the deposition of H3K27Ac and H3K4me3 at the promoters of trained immune genes, allowing for them to be primed for enhanced transcription. The activation of these mechanisms also creates a feed forward loop that allows for training to be self-sustaining and long-lived. The epigenetic marks associated with enhanced transcription of trained immune genes also occur on the promoters of metabolic genes, sustaining the shifted metabolism (6). It has also been recently demonstrated that the accumulation of mevalonate—a metabolite from increased cholesterol metabolism—stimulates the IGF1 receptor in an autocrine way, inducing sustained Akt-mTOR-HIF1α signaling (53). In addition to this, it is thought that the increased cholesterol content plays a role in remodeling the cell membrane to accommodate a greater number of receptors, increasing the sensitivity of the cell to future stimuli.

## The Role of Nuclear Architecture and Long-Non Coding RNAs

A long-standing question in the field of trained immunity involves understanding the molecular mechanisms by which the epigenetic marks are discretely deposited at specific loci at the promoters of trained immune genes. As previously described, the cellular changes that result from the altered metabolism reinforce this process by providing the substrates and co-factors that are needed for epigenetic reprogramming. However, the accumulation of these essential metabolites occurs at a global level in the cell, hinting at the existence of spatially regulated mechanisms in the nucleus to achieve targeted epigenetic changes.

The 3D spatial organization of chromatin is essential for the regulation of gene expression (54). The DNA within the eukaryotic nucleus is compartmentalized into discrete regions of enriched chromosomal contacts, termed topologically associated domains (TADs). Within these TADs, functionally related genes are brought into close proximity with each other, through the formation of chromosomal loops, to facilitate efficient and insulated co-regulation (55). High-throughput chromosomal conformation capture (3C)-derived techniques, such as Hi-C, have revealed that immune genes are segregated into TADs, suggesting an important role for nuclear architecture in the regulation of their transcription (Figures 1C,D) (56, 57). Furthermore, in the last two decades, lncRNAs have been established as functional molecules that position themselves at the nexus of RNA, DNA, and protein interactions to serve as important modulators of gene expression (58). Importantly, it has also been demonstrated that lncRNA activity can be directed to specific loci by the spatial organization of the chromatin (31, 59). Thus, nuclear architecture, together with lncRNA function, could potentially regulate immune gene transcription in a spatially-dependent manner.

One of the most classic examples that demonstrate this are enhancer RNAs (eRNAs) (60–62). These emanate from enhancer regions within the genome and are transcriptionally regulated in a cell-type specific manner to mediate the formation of intra-TAD chromosomal loops through the recruitment of the Mediator complex (60). This allows for enhancer elements, and their associated transcription factors, to be brought into close spatial proximity to their cognate promoter, providing a mechanism for highly controlled spatiotemporal and tissue specific regulation of gene expression (60). This exquisite regulation is thought to play a role in trained immunity. Indeed, a sub-class of enhancers, termed latent enhancers, have been demonstrated to contribute to epigenetic memory. Upon the initial stimulus and the activation of signaling networks, inactive and unmarked enhancers are decorated with transcription factors, which acutely acquire the typical histone modifications (H3K4me1 and H3K27Ac) associated with an active enhancer region. However, subsequent to the removal of the stimulus, many of these enhancers do not return to their latent state, with the H3K4me1 mark in particular, persisting. Therefore, when these cells are restimulated, a much faster and robust enhancer-mediated inflammatory response can be achieved (63).

LncRNAs can also function as molecular scaffolds on which multi-protein complexes can assemble. Furthermore, lncRNAs can serve as guides to direct these complexes to specific gene loci for targeted functionalities. HOTTIP is a prime example that can perform both these functions to epigenetically regulate homeotic gene expression during development. HOTTIP is expressed from the HoxA gene locus and binds to the adapter protein WDR5, which in turn complexes with the MLL methyltransferase to drive H3K4me3 deposition at the promoters of homeotic genes (59, 64). In a recent study, a novel class of lncRNAs, named Immune-gene Priming lncRNAs (IPLs) were discovered to be responsible for the accumulation of H3K4me3 at the promoters of trained immune genes (Figure 1D) (31). Through a bespoke bioinformatic pipeline, it was revealed that emanating from within each of the TADs housing key trained immune genes, was a single lncRNA transcript that interacted with the WDR5/MLL complex to direct local H3K4me3 accumulation. In depth mechanistic studies on a candidate IPL found in the important ELR+ CXCL TAD (containing IL8, CXCL1, CXCL2, and CXCL3), named UMLILO (**u**pstream **m**aster **l**ncRNA of the **i**nflammatory chemokine **lo**cus), demonstrated that the lncRNA was not involved in establishing the local chromatin contacts within this TAD, but rather exploited pre-formed three-dimensional topology for its function. Strikingly, ablation of the UMLILO transcript disrupted β-glucan training of the ELR+ chemokines in human monocytes. Furthermore, it was observed that the murine CXCL TAD, which lacks UMLILO, is incapable of being trained, presumably because of its absence. Targeted insertion of UMLILO into the syntenic mouse locus allowed for murine CXCL genes to gain the ability to be trained upon exposure to β-glucan. The study also demonstrated that the expression of UMLILO was dependent on the β-glucan-induced, calcium-dependent nuclear translocation of NFAT, suggesting that the initial immune pathogen receptor (PRR) signaling is important to activate IPL expression for immune gene training. In short, this study firmly establishes an essential role of lncRNAs in trained immunity by revealing the molecular mechanism by which H3K4me3 is discretely accumulated at the promoters of immune genes that are trained. This demonstrates the central role of IPLs in trained immunity, as all the signaling and metabolic changes converge upon the nucleus to activate the transcription of IPLs and support their function. Furthermore, expression of IPLs could be responsible for the persistence of epigenetic modifications in trained progenitor cells, allowing for the epigenetic inheritance of trained immunity.

The long-lived accumulation of the H3K4me3 mark has also been observed to occur on the metabolic genes of trained monocytes (6). The epigenetic priming of these genes is most likely essential to maintaining the shifted metabolic status that supports the trained phenotype. We suspect that the accumulation of H3K4me3 on these metabolic genes is mediated by a family of lncRNAs that is functionally similar to the IPLs and are equally as important in achieving and sustaining the trained state. The elucidation of these lncRNA candidates in future work would involve applying the bioinformatic pipeline described by Fanucchi et al. (31) to TADs with metabolic genes associated with trained immunity. This could potentially reveal another novel class of metabolic gene priming lncRNAs (MLPs) that are responsible for the epigenetic modifications observed at the promoters of metabolic genes involved in trained immunity.

It is clear that the description of the protein complex associated with the IPLs (31) in some aspects is incomplete, and not limited functionally to histone methyltransferase activity. Further in depth analysis of the IPL protein binding partners could reveal other proteins that expand the functional repertoire of the IPL ribonucleoprotein complex. For example, a metabolic complex comprising of pyruvate kinase M2 (PKM2) and pyruvate dehydrogenase (PDC), which together regulate the production of acetyl-CoA, was found to interact with the p300 histone acetyltransferase to epigenetically regulate the expression of the aryl hydrocarbon receptor (AhR) (65). It was demonstrated that the activity of p300 was dependent on the local production of acetyl-CoA by PKM2 and PDC. In another example, methionine adenosyltransferase (MAT), which is responsible for the synthesis of the methyl donor for histone methylation, *S*-adenosylmethionine (SAM), complexed with the H3K9 methyltransferase SETDB1 (66). Together, this complex was shown to regulate the *COX-2* locus by producing both the methyl donor substrate and depositing the repressive H3K9me3 mark. These examples highlight the intimate relationship between cellular metabolic machinery and epigenetic modulators. By forming such complexes, changes in the local concentration of metabolites can be “sensed” to create stricter spatial limitations for discrete epigenetic changes to occur at the relevant genes. It is possible that such complexes function in conjunction with “IPL-like” lncRNAs, further enhancing the specificity and efficiency of targeted epigenetic modifications.

## An Opportunity for Novel Immunotherapies

The discovery of innate immune memory has fundamentally changed the way we understand the immune system. The line that once clearly divided innate and adaptive immunity is now more blurred than ever before as we continue to uncover the intricacies of the immune system and how each of these arms feed into each other's function. The inflammatory response is the most rapid and effective form of defense against acute pathogenesis and injury. However, its dysregulation or prolonged activation is known to contribute to a host of non-infectious and chronic diseases, ranging from auto-inflammatory disorders, such as arthritis, to cancer. This has led to intense research efforts for the development of immunotherapies with which to modulate the inflammatory response. Trained immunity has been of particular interest as it presents a way to educate innate immune cells and fine tune their inflammatory response. Despite the benefits of trained immunity in immunological protection, it is also thought that the enhanced inflammatory response may undesirably contribute to the development and progression of diseases characterized by excessive inflammation (67). In many of these diseases, innate immune cells display alterations in cellular metabolism, epigenetic changes and enhanced inflammatory responses, all of which are traits of trained immunity (53, 67). Therefore, the ability to control and curb the effects of trained immunity would be desirable in such cases. Conversely, in cancer, tumor-associated macrophages (TAMs) are reprogrammed by the tumor micro-environment to become immunosuppressive of the T-cell mediated anti-tumor responses (68). Therapeutics that are able to kick-start the immune responsive activity of TAMs could be highly beneficial for the treatment of cancer. For several years, BCG has been used as an immunotherapy adjuvant for the treatment of bladder cancer (69). Indeed, it has already been demonstrated in a few studies where β-glucan treatment reversed the immunosuppressive state of TAMs and delayed tumor progression (70, 71).

Our current understanding of the molecular mechanisms behind trained immunity has already revealed numerous immunological, metabolic, and epigenetic targets that could be exploited to inhibit or potentiate trained immunity for therapeutic purposes (20, 21, 34, 44, 47, 72). However, the function of many of these are not exclusively limited to trained immunity and generally serve in various other cellular processes, potentially leading to drug related side-effects and cellular toxicity. With the recent insights into the integral role of lncRNAs in the acquisition of epigenetic memory, the next generation of immunotherapies could be directed to modulating the activity of these transcripts, enabling highly specific, durable, and discrete tuning of the trained immune response.

**Figure 2:**
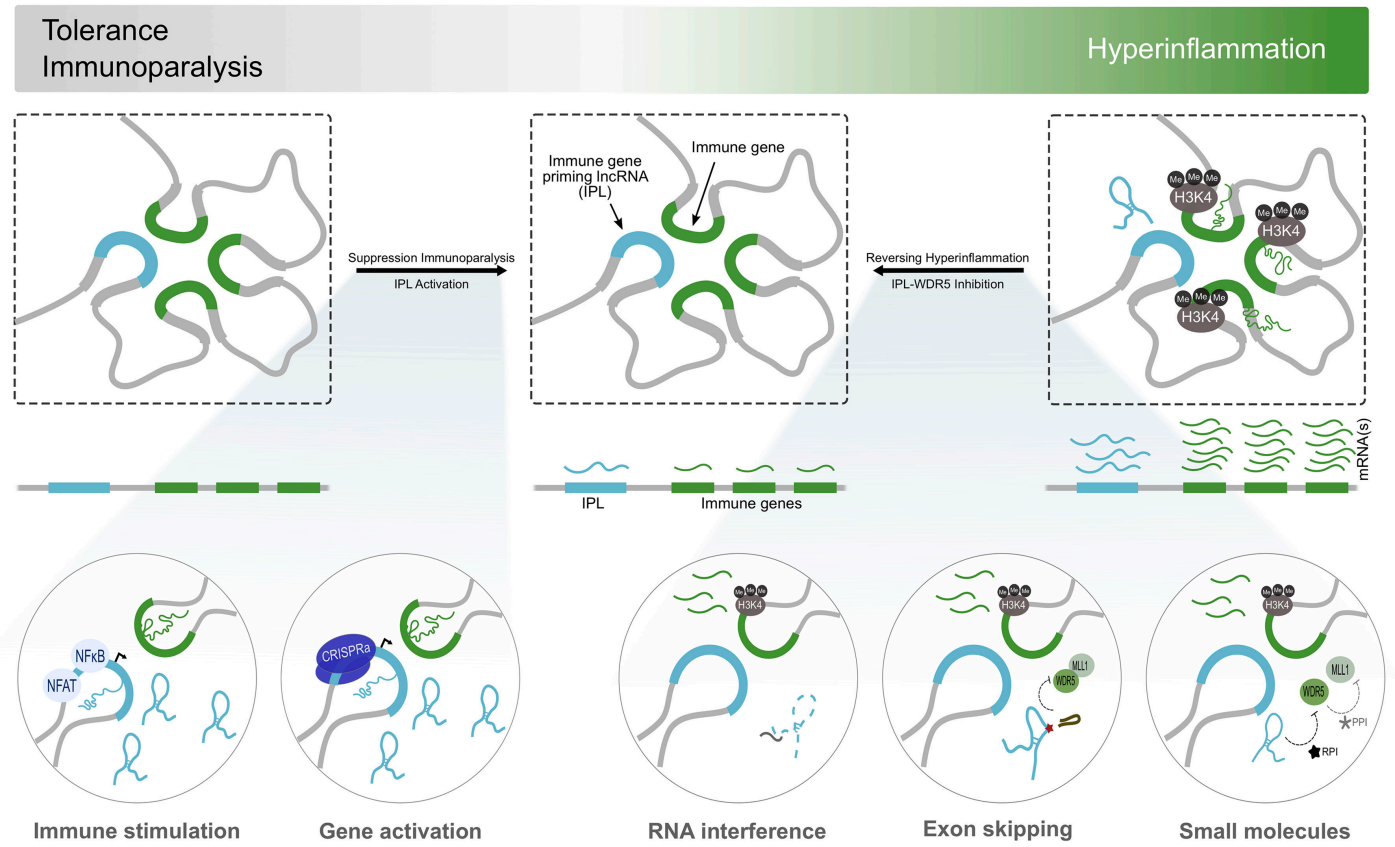
Immunomodulation by IPL-targeted therapeutic approaches. Considering the dysregulation of inflammatory processes contribute significantly to the establishment of many diseases, regulators of the immune transcriptional response, such as IPLs, become attractive therapeutic targets. States of immunoparalysis, characterized by the absence of the H3K4me3 mark and the refractory activation of immune gene transcription, can be reversed by immune stimulation using BCG or β-glucan. This initializes transcriptional programmes involved in trained immunity to kickstart the inflammatory response. Alternatively, the direct and discrete activation of IPL expression using CRISPRa could lead to the recruitment WDR5/MLL to deposit H3K4me3 at the promoters of immune genes to reverse their transcriptional paralysis. On the other side of the spectrum of inflammation resides the continuous activation of immune genes and accumulation of H3K4me3 marks at their promoters. Interfering with the expression or function of the IPLs (RNA interference, exon skipping, ribonucleoprotein inhibitors) results in the local deprivation of H3K4me3, curbing the transcriptional activity of the immune genes, and overall, the inflammatory response.

The discovery and development of small molecule inhibitors which occlude the necessary interactions within the ribonucleoprotein complex or grossly contort the lncRNA structure, could serve as ways to inhibit IPL function (Figure 2). For example, MM102, a small molecule inhibitor of the WDR5/MLL1 interaction, reduces chemokine expression by interfering with IPL ribonucleoprotein assembly and function (31, 73, 74). The activity of such molecules often acts indiscriminately on all cell types, necessitating the need to develop conjugated molecules or drug carriers that function as homing ligands to cell-specific receptors. Furthermore, the emergence of large screening and drug repurposing efforts will also aid the discovery of novel small molecules that can be safely used as IPL inhibitors. Classical RNA-targeting modalities, such as RNA interference (RNAi) and antisense oligonucleotides (ASOs), can also inhibit lncRNA function by depleting the cellular concentrations of the transcript in a sequence dependent manner (Figure 2). It is however important to consider the subcellular localization of the lncRNA when applying these RNA targeting modalities (75). ASOs might be better suited for the knockdown of nuclear-resident lncRNAs, such as the IPLs, as the mechanisms of ASO function (transcript degradation or exon skipping) occur in the nuclear compartment (75). There is documented success using small-interfering RNAs (siRNAs) and short-hairpin RNAs (shRNAs) for targeting lncRNAs despite the controversy surrounding the activity of RNAi in the nucleus (76–78). However, differences in efficacy and dosages between ASOs and RNAi for IPL inhibition, as a result of the subcellular localization of their function, may become apparent when administering these molecules as therapeutics at a systemic level. Furthermore, the efficient and targeted delivery of ASOs and siRNAs remains as one of the central challenges encumbering their use as a therapy. Efforts to improve this have made use of functionalized nanoparticles, stabilizing chemical modifications and cell-type specific ligand conjugates to improve efficacy, efficiency and specificity of delivery and reduce immunogenicity (79, 80). shRNAs can be delivered using viral vectors, such as recombinant adeno-associated viruses (rAAVs) programmed to have cell-type specific tropisms (81–83). These strategies are already being implemented in numerous clinical trials for the knockdown of disease causing mRNAs, highlighting the potential use of this technology for targeting lncRNAs (79). Furthermore, by knocking down lncRNAs that exert their function as epigenetic regulators of mRNA expression, a significant barrier to the activation of mRNA transcription is introduced. This implies that targeting lncRNAs will be more therapeutically durable and efficacious, compared to the knockdown of mRNAs.

The development of therapeutic strategies for the discrete activation of lncRNAs is inchoate. Perhaps the most promising tool for this is the use of transcriptionally activating CRISPR/dCas9 to potentially switch on and overexpress IPLs to reverse states of immunotolerance (Figure 2) (84). However, this technology is still in its infancy and experiences difficulties associated with delivering the CRISPR/dCas9 genetic payloads and the potential for off-target gene activation, preventing its application as a safe and viable therapeutic.

As our appreciation for the role of lncRNAs in gene regulation expands, we anticipate that these transcripts will become highly desirable novel drug targets for augmenting or suppressing gene transcription with extreme specificity and long-lasting effects. With this, the technology to control the expression and perturb the function of these biomolecules will advance to become the next generation of therapeutics in the arsenal of modern medicine.

## Conclusion and Future Perspectives

In this brief review we present a progression of molecular mechanisms that lead to the establishment of trained immunity, from cell metabolism to epigenetics. We highlight the convergence of various signaling pathways and metabolic changes that manifest as discrete epigenetic changes on the promoters of trained immune genes through the function of a novel class of lncRNAs. The process of this epigenetic memory formation begins with a training stimulus activating various PRR signaling pathways to induce transcription factor-based changes in gene expression. This involves the activation of immune genes and lncRNAs, of which some of the most critical for training are the recently discovered IPLs. Concurrent to this, is the activation of metabolic genes responsible for enhanced glycolysis, glutaminolysis, and cholesterol synthesis. The resultant metabolic changes are essential in preparing the cell for training, as they provide the energy and metabolites required for the epigenetic reprogramming and the anticipated enhanced transcription. Central to the entire training process is the expression and activity of the IPLs, which direct epigenetic modulators to the trained immune genes for the discrete and sustained accumulation of the H3K4me3 mark. Ultimately, the progression of these events leads to enhanced gene transcription and a more robust secondary inflammatory response. In this review, we describe the mechanisms of trained immunity associated with β-glucan trained monocytes, as this has been the standard model to study this phenomenon. However, we are aware that many cells types of the myeloid lineage can also be trained, and with numerous different stimuli. We would expect that many of these mechanisms are conserved in different cell types, with some variation, to establish innate immune memory.

With the rapid advancements in the fields of immunology, metabolism and transcription, deeper insights are being gained into how each of these facets of cell biology coalesce to “write” the epigenetic memories associated with training. With an ever more granular description of the progression of molecular events that lead to trained immunity, various mechanisms that regulate the trained response can be identified as targets for novel therapies. The discovery of the central role of lncRNA function in the establishment of trained immunity has revealed a novel class of therapeutic targets for potentially controlling inflammatory responses in a very discrete manner. Furthermore, as the catalog of functionally annotated lncRNA transcripts grow, we foresee more lncRNA classes that are important for the regulation of trained immunity and inflammation will be revealed.

## Author Contributions

ETF and LD discussed, planned, and wrote the review. SF and MMM oversaw the writing process, discussed, and edited the review.

### Conflict of Interest Statement

The authors declare that the research was conducted in the absence of any commercial or financial relationships that could be construed as a potential conflict of interest.
